# Variable Cyanobacterial Toxin and Metabolite Profiles across Six Eutrophic Lakes of Differing Physiochemical Characteristics

**DOI:** 10.3390/toxins9020062

**Published:** 2017-02-10

**Authors:** Lucas J. Beversdorf, Chelsea A. Weirich, Sarah L. Bartlett, Todd. R. Miller

**Affiliations:** 1 Joseph J. Zilber School of Public Health, University of Wisconsin-Milwaukee, Milwaukee, WI 53211, USA; lucasb@uwm.edu (L.J.B.); cweirich@uwm.edu (C.A.W.); 2 School of Freshwater Sciences, University of Wisconsin-Milwaukee, Milwaukee, WI 53204, USA; bartle34@uwm.edu

**Keywords:** microcystin, anabaenopeptin, cyanopeptolin, nodularin, microginin, anatoxin, cylindrospermopsin, saxitoxin

## Abstract

Future sustainability of freshwater resources is seriously threatened due to the presence of harmful cyanobacterial blooms, and yet, the number, extent, and distribution of most cyanobacterial toxins—including “emerging” toxins and other bioactive compounds—are poorly understood. We measured 15 cyanobacterial compounds—including four microcystins (MC), saxitoxin (SXT), cylindrospermopsin (CYL), anatoxin-a (ATX) and homo-anatoxin-a (hATX), two anabaenopeptins (Apt), three cyanopeptolins (Cpt), microginin (Mgn), and nodularin (NOD)—in six freshwater lakes that regularly experience noxious cHABs. MC, a human liver toxin, was present in all six lakes and was detected in 80% of all samples. Similarly, Apt, Cpt, and Mgn were detected in all lakes in roughly 86%, 50%, and 35% of all samples, respectively. Despite being a notable brackish water toxin, NOD was detected in the two shallowest lakes—Wingra (4.3 m) and Koshkonong (2.1 m). All compounds were highly variable temporally, and spatially. Metabolite profiles were significantly different between lakes suggesting lake characteristics influenced the cyanobacterial community and/or metabolite production. Understanding how cyanobacterial toxins are distributed across eutrophic lakes may shed light onto the ecological function of these metabolites, provide valuable information for their remediation and removal, and aid in the protection of public health.

## 1. Introduction

Cyanobacteria represent an ancient and diverse phylum of photosynthetic bacteria that inhabit ubiquitous, seemingly disparate ecosystems. Not surprisingly then, cyanobacteria have adapted unique evolutionary strategies to survive, compete, and even dominate various environments. In particular, cyanobacteria may be most infamous in eutrophic lakes, where large accumulations of noxious biomass can greatly reduce water clarity and quality, disrupt food webs, decrease biodiversity, and affect animal and human health. In addition, cyanobacteria have evolved to produce hundreds of secondary metabolites [1] that may or may not be toxic to humans, but for many of these compounds, as well as the species that produce them, their occurrence and distribution in freshwater lakes is unknown. For many of these toxins, the biosynthetic pathways have been determined, which has not only accelerated our ability to detect and quantify their presence in environmental systems (reviewed in [2]), it has laid the ground work for more mechanistic studies to be performed. However, the eco-physiological role of these metabolites remains elusive, the environmental controls on production are not well constrained, and the indirect effects they may have on the microbial loop, food web, and/or whole ecosystem are widespread. Because climate projections suggest that the frequency and extent of these harmful cyanobacterial blooms will increase in the future [3,4], including the risk they pose to human health, there is an immediate need to identify and quantify these metabolites in freshwaters, especially water bodies that may be used as recreational and drinking water sources.

Many of the known cyanobacterial secondary metabolites share structural similarities and functional properties and thus, can be categorized into specific classes (reviewed in [5]). The two most abundant by structural class are the peptides and alkaloids. The most notable peptide is the hepatotoxin, microcystin (MC), which is ubiquitous in eutrophic lakes and the most observed cyanobacterial toxin worldwide [6]. In animals and humans, MC binds to protein phosphatases 1 and 2A [7] leading to acute liver necrosis and death [8,9] and may contribute to other diseases [10,11,12]. In addition, MC is a known carcinogen [13,14,15] and has been implicated in long-term diseases such as colorectal and liver cancer in the United States, China, and Serbia [16,17,18,19]. Many of the other compounds in the peptide class, which are also presumably highly abundant in nature—such as the cyanopeptolins, microginins, and anabaenopeptins—are considered “nontoxic.” Little is known about the potential health effects of these compounds on animals and humans and is mostly speculative based on a few preliminary experiments. For example, the bioactive compound, cyanopeptolin (Cpt) 1020, has exhibited neurotoxic effects in one zebra fish study [20], and Gademann et al. [21] found Cpt1020 to be toxic to the freshwater crustacean, *Thamnocephalus platyurus*. They further suggest that Cpt1020 be considered as important as other acutely acting toxins such as MCs due to its trypsin inhibiting characteristics. Microginin (Mgn) 690 has been shown to inhibit angiotensin-converting enzyme [22] and has been proposed to treat high blood pressure [23]. Thus, while not toxic, Mgn690 has been investigated for its pharmaceutical characteristics. Conversely, some compounds have yet to show any human effects despite having comparable mechanisms of other toxins [24]. For example, while anabaenopeptin (Apt) F is a known protease inhibitor, it has also been shown to inhibit protein phosphatases like MCs [25]. If nontoxic, these compounds may still be extremely important ecologically. AptB and F have been shown to induce lysis of the cyanobacteria, *Microcystis aeruginosa* [26], which may have profound impacts on cyanobacterial community dynamics and the release of other toxins into surface waters. Other toxic, but less prevalent (for now) peptides of interest in include nodularins (NOD). NOD, which is structurally very similar to MC, is also a protease inhibitor [27]; however, in aquatic systems, NOD has been shown to be produced only by the cyanobacteria *Nodularia*, and with the exception of one isolated freshwater strain [28], has been mostly associated with brackish waters [29]. Similarly, cylindrospermopsin (CYL) has been observed in both freshwater and brackish waters and until recently, has been considered a subtropical toxin.

CYL is an alkaloid, structurally the second most common group of cyanobacterial toxins [5]. It is produced by *Cylindrospermopsis raciborskii*, among others, but recent reports suggest *C. raciborskii* may be invasive due to several physiological adaptations and warming temperatures [30], and thus, the distribution of *C. raciborskii* and CYL, may spread to temperate lakes. CYL, an analog of uracil, is a hepatotoxin that inhibits protein synthesis, induces oxygen radical formation, and causes DNA strand breaks, all of which lead to liver and kidney toxicity [31,32]. Other commonly observed alkaloids include the neurotoxins anatoxin-a, homo-anatoxin-a, anatoxin-a(S), and saxitoxins. Anatoxin-a (ATX) and homo-anatoxin-a (hATX) mimic the neurotransmitter acetylcholine and bind irreversibly to nicotinic acetylcholine receptors in peripheral nerve cells [33]; the eventual result of ATX poisoning is respiratory paralysis and death. Anatoxin-a(S), which is more potent than ATX, inhibits the breakdown of acetylcholine and other choline esters leading to continuous peripheral nerve cell stimulation [34]. Due to lack of certified reference material, few studies have been able to measure anatoxin-a(S) in the environment. Saxitoxin (SXT), one of a group of structurally related compounds known as paralytic shellfish poisons (PSPs), binds to sodium-gated channels [35], as well as potassium and calcium associated channels [36], leading to paralysis, cardiac arrest, and death. Many other alkaloids are not toxic, but instead, are antimicrobial. In particular, several indole alkaloids act as anti-fungal and anti-algal agents. Another cyanobacterial alkaloid that could be important ecologically is nostocarboline, which is an anti-cyanobacterial/algal compound [37]. Interestingly, nostocarboline is also an inhibitor of acetylcholinesterase and trypsin [38].

Much is known about the distribution of more common toxins (e.g., MCs), but there is little information about the distribution of multiple classes of cyanobacterial metabolites, including emerging toxins and other bioactive compounds, across lakes. In addition, it is not clear how mixtures of these bioactive compounds interact and differ across lakes that commonly experience cyanobacterial blooms. To that end, we measured 15 cyanobacterial compounds—nine of which are known toxins and six that are considered bioactive compounds or emergent toxins—in six eutrophic lakes over the course of six months. To our knowledge, few studies have performed such a quantitative, spatiotemporal survey with this number of cyanobacterial toxins/bioactive compounds and lake parameters. Our goal was to determine the distribution of these compounds across a suite of eutrophic lakes that regularly experience similar noxious cyanobacterial blooms, but vary in physical and chemical characteristics.

## 2. Results

**Figure 1:**
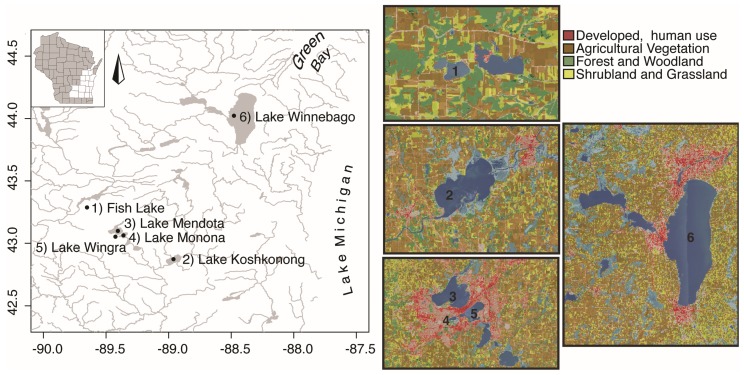
(**Left**) Map of six study lakes located in Central and South Central Wisconsin, USA; and (**Right**) United States Geological Survey (USGS) topographical maps of land use for each lake in this study. Numbers on the right correspond to associated lake on the left.

We chose these lakes because all six: (1) are eutrophic and have experienced noxious cyanobacterial blooms; (2) differ physically (e.g., size and depth); (3) have variable land use and watershed size; and (4) are recreational lakes with Lake Winnebago serving as a drinking water source for approximately 300,000 people (Figure 1, Table 1). In addition, four of these lakes (Fish, Mendota, Monona, and Wingra) are sampled regularly as part of the North Temperate Lakes-Long Term Ecological Research (NTL-LTER) program providing historical trends in water quality. 

### 2.1. Metabolite Detects and Ranges Observed

**Figure 2:**
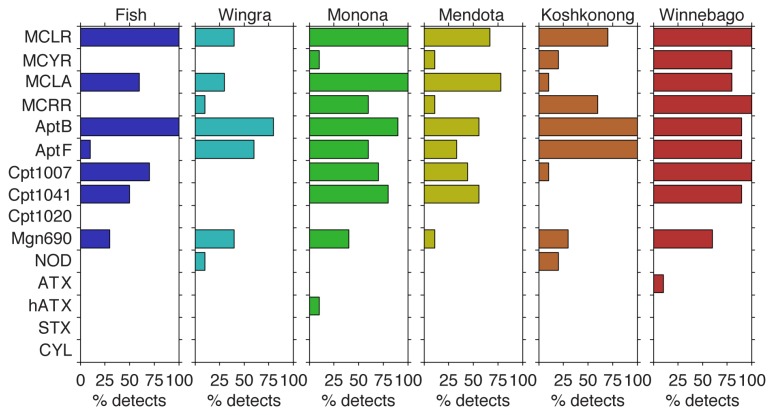
Percent detects of 15 metabolites surveyed in this study for each lake. MC = microcystin; Apt = anabaenopeptin; Cpt = cyanopeptolin; Mgn = microginin; NOD = nodularin; ATX = anatoxin-a; hATX = homo-anatoxin-a; STX = saxitoxin, CYL = cylindrospermopsin. Cpt1020, STX, and CYL were not detected in this study. *n* = 10 for all lakes except Lake Mendota, where *n* = 9.

Cyanobacterial metabolites were detected in all six lakes with 12 of the 15 metabolites being detected at least once. The bioactive peptide AptB was detected more than any other metabolite measured, occurring in 85.9% of samples (*n* = 59) (Figure 2), while AptF was observed in 58.9% of samples. MCLR is often the most common MC variant reported worldwide. Here, MCLR was detected in 79.5% of the total samples and in 100% of the samples collected from Lake Winnebago, Lake Monona, and Fish Lake. MCLA, MCRR, and MCYR were detected less frequently at 59.6%, 40.2%, and 20.2% of total samples, respectively. The bioactive metabolite and possible neurotoxin Cpt1020 was not detected in any samples, but the structurally similar compounds Cpt1007 and Cpt1041 were detected in 49.1% and 45.9% of samples, respectively. CYL and SXT were also not detected in any of the lakes. However, ATX and hATX were detected once each in Lake Winnebago and Lake Monona, respectively. Interestingly, NOD, which is normally associated with brackish waters, was detected in 5% of samples with all NOD detects coming from the two shallowest lakes—Wingra and Koshkonong. The linear peptide Mgn690 was detected in 35.2% of samples.

**Figure 3:**
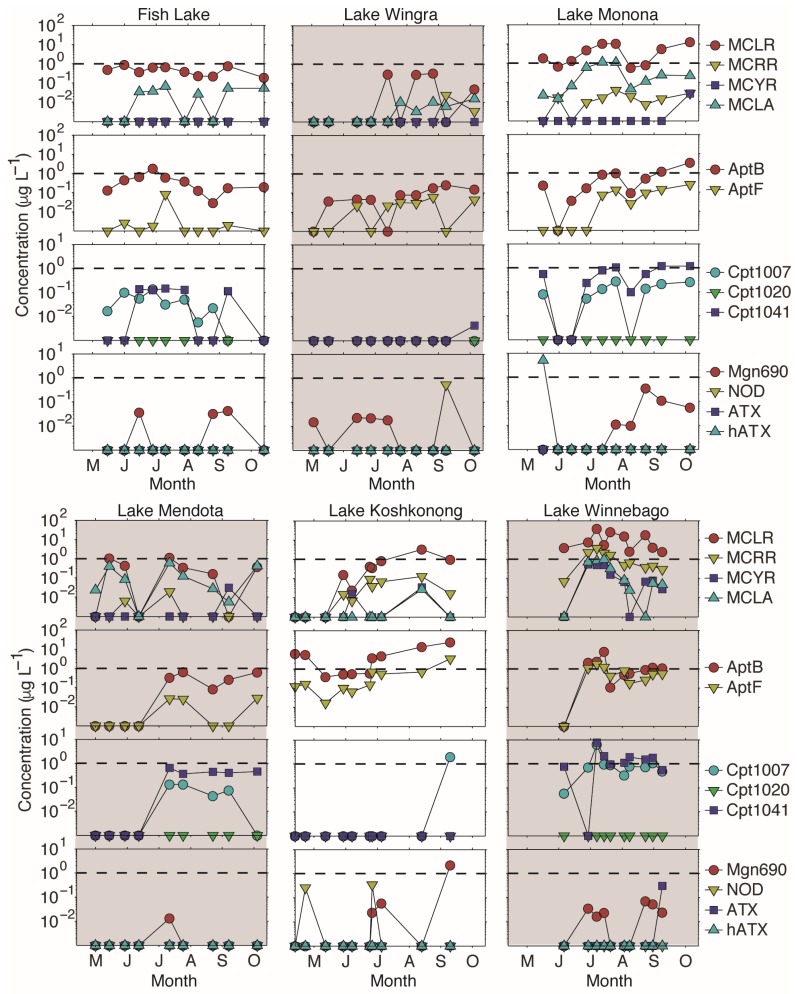
Concentrations of all metabolites detected in this study. The dotted line represents a concentration of 1 µg·L^−1^, the World Health Organization level for safe drinking water for microcystin, anatoxin-a, and cylindrospermopsin. MC = microcystin; Apt = anabaenopeptin; Cpt = cyanopeptolin; Mgn = microginin; NOD = nodularin; ATX = anatoxin-a; hATX = homo-anatoxin-a.

Despite the large number of detects for the majority of these compounds, concentrations were highly variable. For example, MCLR was detected in 100% of samples in Fish Lake, but the Total MC (i.e., sum MCLR, MCLA, MCRR, and MCYR) concentration was never greater than 1.0 µg·L^−1^ (Figure 3, Table 2). Conversely, for every Lake Winnebago sample, the MCLR concentration was greater than 1.0 µg·L^−1^, with a mean Total MC of 13.9 µg·L^−1^. Similarly, AptB and AptF were detected in 80% and 60%, respectively, of Lake Wingra samples and never exceeded 1.0 µg·L^−1^. However, 60% of all Lake Koshkonong samples had Total Apt concentrations greater than 4.0 µg·L^−1^, with AptB and AptF being detected in 100% of samples. Similar to MCs, Cpts were detected frequently in Fish Lake and Lake Mendota, but concentrations never exceeded 1.0 µg·L^−1^. Total Cpt was detected most frequently in Lakes Monona and Winnebago, and was greater than 1.0 µg·L^−1^ in 80% of Lake Winnebago samples. While hATX was only detected once, it was detected at 5.1 µg·L^−1^. The max Mgn690 concentration observed was 2.2 µg·L^−1^ in Lake Koshkonong.

### 2.2. Intra-Lake Differences in Observed Metabolites

We first used a Lilliefors Test to determine if lake metabolites were normally distributed. Of the 12 metabolites detected—including Total MC, Total Apt, and Total Cpt—none of the observations were significantly (*p* < 0.05) normally distributed following a post hoc Monte Carlo simulation. Therefore, we used a Spearman rank correlation to compare metabolite concentrations within lakes. Not surprisingly, in most cases, congeners of the same metabolite structural group correlated to each other (e.g., AptB correlated to AptF) except for metabolites that had concentrations at or near detection for the entire sampling season. However, very few correlations existed between individual congeners of other metabolite classes. In Lake Mendota, Total Apt and Total Cpt were significantly correlated (*p* < 0.05, Rho = 0.84). In Lake Monona, Total Apt/Total Cpt (*p* < 0.05, Rho = 1.00), Total Apt/Total MC (*p* < 0.05, Rho = 0.82), and Total MC/Total Cpt (*p* < 0.05, Rho = 0.81) were all significantly correlated to each other, and in Fish Lake, Total Apt was significantly correlated to Total Cpt (*p* < 0.05, Rho = 0.75). All of the above significant correlations were positive. None of the Spearman rank correlations in Lake Wingra were significant, likely due to the very low metabolite concentrations. While these correlations among metabolite classes existed within lakes, the same correlations were not consistent across lakes.

### 2.3. Inter-lake Differences in Observed Metabolites

**Figure 4:**
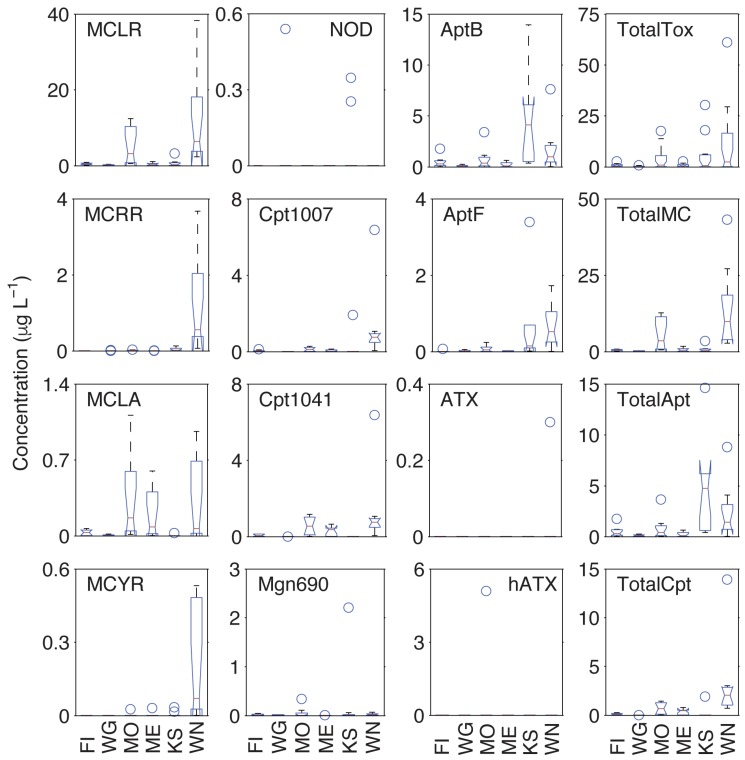
Kruskal–Wallis box and whisker plot results of lake metabolite concentrations between lakes in this study. The central line represents the median. The top and bottom of the box represents the 25th and 75th quartiles, respectively. The whiskers extend to data points that are not considered outliers, which is ±2.7 standard deviation from the mean, and plus symbols are outliers. Notches that do not overlap represent significant differences in metabolite distributions. FI = Fish Lake; WG = Lake Wingra; MO = Lake Monona; ME = Lake Mendota; KS = Lake Koshkonong; WN = Lake Winnebago.

To compare between lake metabolite concentrations, we used a Kruskal–Wallis test, which uses the median concentration of each group rather than the mean as the metric of statistical difference, followed by a post hoc analysis using the Dunn–Sidak method [39]. We used this test because none of the lakes had normal distributions of metabolites (see Table 2 for mean, median, and max concentrations of each metabolite for each lake). Lake Winnebago had the largest concentration of Total MCs and was significantly greater (*p* < 0.05) than all other lakes except Lake Monona, which had Total MC concentrations significantly greater than Lake Koshkonong and Lake Wingra (Figure 4). These differences were mostly a result of MCLR, MCRR, and MCYR concentrations. The distribution of MCLA across the six lakes was very similar. Similarly, Lake Winnebago had the largest concentration of Total Cpt, which was significantly greater (*p* < 0.05) than all lakes except Lake Monona (Figure 4). Lake Mendota had the third highest median Cpt levels but was not significantly greater than Fish Lake, Lake Wingra, or Lake Koshkonong; both congener, Cpt1007 and Cpt1041, concentrations were greatest in Lake Winnebago. Total Apt concentrations were greatest in Lake Koshkonong and Lake Winnebago (Figure 4); both lakes had Apt concentrations significantly greater (*p* < 0.05) than Lakes Wingra and Mendota. Fish Lake and Lake Monona Apts were not significantly greater than Lakes Wingra or Mendota, nor were they significantly less than Lakes Koshkonong and Winnebago.

**Figure 5:**
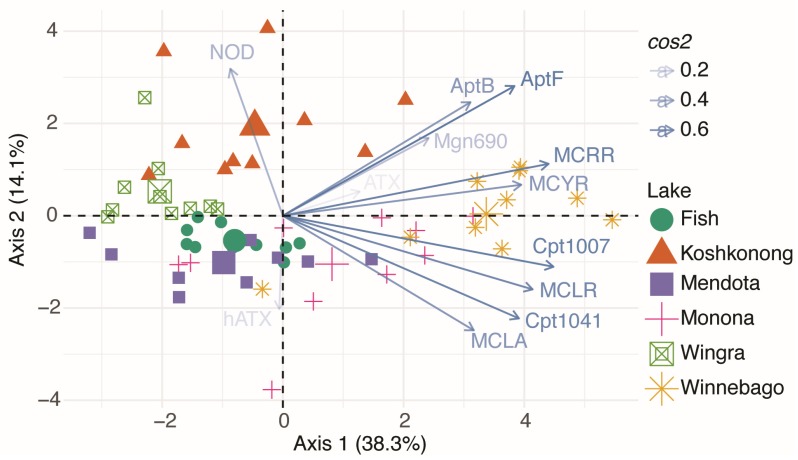
Principal component analysis (PCA) of lake metabolite profiles observed in this study. Each point represents all metabolites measured in a particular sample. Samples are separated by Euclidean distance where the closer samples are to each other, the more similar their metabolite profiles are. Lakes were significantly different based on analysis of similarity (ANOSIM; *p* < 0.05; *R* = 0.55). R values represent how similar samples are. For example, an R value of 1 represents samples that are completely unique, whereas an R value of 0 represent samples that are identical. Arrows point in the direction of samples with higher correlations and the length of each arrow represents the magnitude of that correlation squared (*cos*2). For example, cyanopeptolin 1007 (Cpt1007) had the strongest influence on samples collected from Lake Winnebago. The largest symbol in each group represents the centroid of all samples from that lake.

No significant differences existed for NOD, ATX, and hATX due to the infrequent detects and low concentrations. Interestingly, total metabolite concentrations (Figure 4, “TotalTox”) were also not significantly different (*p* > 0.17), based on non-parametric analysis, between any of the lakes despite significant differences in MC, Apt, and Cpt concentrations. In order to investigate differences in lake metabolite profiles, we performed a principal component analysis (PCA) between lakes, and not surprisingly, samples grouped by lake based on metabolite profile (Figure 5). These differences were significant based on an analysis of similarity (ANOSIM; *p* < 0.001; R = 0.55). Thus, the milieu of cyanotoxin metabolite mixtures varied significantly between lakes, even lakes of similar trophic status. The importance of each lake or variable in this analysis was defined by *cos*2, which represents the quality of representation of each variable and is equivalent to the square of the correlation coefficients for each sample. For example, Lake Winnebago had the greatest overall contribution to the variability of the first axis (*cos*2 = 6.15), which was most influenced by Cpt1007 (*cos*2 = 0.71), MCRR (*cos*2 = 0.69), and MCLR (*cos*2 = 0.61) (Figure 5, Table S1). Conversely, Lake Koshkonong had the greatest contribution to the variability of the second axis (*cos*2 = 3.08), which was most influenced by NOD (*cos*2 = 0.36), AptF (*cos*2 = 0.28), and MCLA (*cos*2 = 0.22). AptB and MCLR contributed greatly to both axes as they were the most frequently detected compounds overall.

### 2.4. Physiochemical Characteristics

To explore the spatial differences in lakes further, we compared lake size and depth, chlorophyll, and nutrient concentrations. We do not make direct correlations between these variables and metabolite concentrations because not all samples were collected on the same day, and limited nutrient data exist for Lake Winnebago and Lake Koshkonong. Instead, here we make qualitative comparisons between these lakes and their associated metabolite profiles.

**Figure 6:**
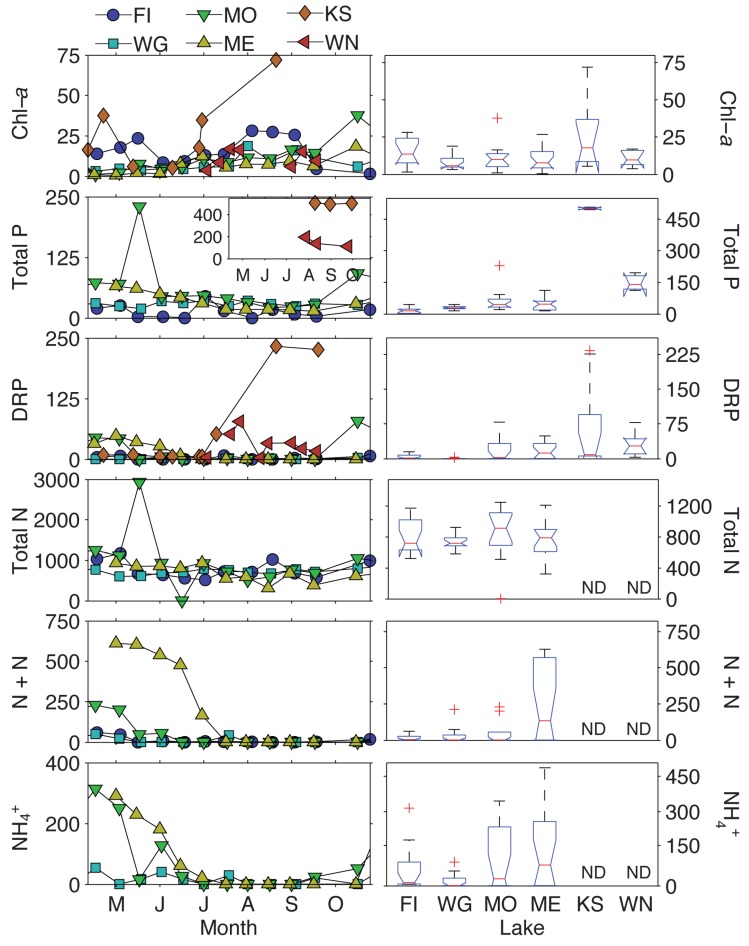
(**Left**) Temporal trends in chlorophyll-*a* and nutrient concentrations observed in this study. (**Right**) Kruskal–Wallis box and whisker plot results of chlorophyll-*a* and nutrient concentrations between lakes. The central line represents the median. The top and bottom of the box represents the 25th and 75th quartiles, respectively. The whiskers extend to data points that are not considered outliers, which is ±2.7 standard deviation from the mean, and plus symbols are outliers. Notches that do not overlap represent significant differences in metabolite distributions. Note that for Lake Koshkonong and Lake Winnebago, *n* = 3 for total phosphorus. Chl-*a* = chlorophyll-*a*; total N = total nitrogen; total P = total phosphorus; N + N = nitrate + nitrite; DRP = dissolved reactive phosphorus; NH_4_^+^ = ammonium. FI = Fish Lake; WG = Lake Wingra; MO = Lake Monona; ME = Lake Mendota; KS = Lake Koshkonong; WN = Lake Winnebago; ND = no data.

Lake Koshkonong had the highest mean chlorophyll-*a* (chl-*a*) concentration (27.2 µg·L^−1^), followed by Fish Lake (14.7 µg·L^−1^), but no significant differences existed between lakes (Figure 6; Table 3). Lake Koshkonong also had the highest mean total phosphorus (TP) concentration (501 µg·^L−1^), followed by Lake Winnebago (149 µg·L^−1^) and Lake Monona (62.4 µg·L^−1^), all of which were significantly different from each other. Dissolved reactive phosphorus (DRP) was also highest in Lake Koshkonong, which increased greatly from near detection in May-July to over 200 µg·L^−1^ in late August (Figure 6). Nitrogen (N) and pH were only measured in Lakes Fish, Wingra, Monona, and Mendota. In these lakes, total N (TN) was not significantly different in any of the lakes. However, nitrate + nitrite (N + N) was significantly greater in Lake Mendota with a mean concentration of 267 µg·L^‑1^. Lake Mendota also had the highest mean ammonium concentration (126 µg·L^−1^). No significant differences existed between the lakes with regard to TN:TP ratios. In addition, very low DRP concentrations led to highly variable dissolved inorganic N (DIN):DRP ratios. For example, in Fish Lake, there were several days where DRP was near detection while N concentrations were comparatively high resulting in DIN:DRP ratios > 1000 and with a variance > 10^6^. If we compare just the medians, Lake Monona had considerably lower DIN:DRP ratios (median = 9.0), suggesting this lake may actually be N limited. Lake pH was virtually identical in these lakes with mean values ranging from 8.49 (± 0.59) to 8.57 (± 0.41). Surface water temperatures were gathered from nearby stations and were not significantly different with means ranging from 23.0 °C (± 3.7) to 25.6 °C (± 1.7). No further analyses were performed with surface water temperature since it auto-correlated with day number.

Despite individual similarities in chl-*a*, nutrients, pH, and temperature, lakes did group differently by their physiochemical differences as variables such as surface area differed by four-fold and maximum depths ranged 2.1–25.3 m. We performed two PCAs to help differentiate some of these lake characteristics (Figure S1): (A) all six lakes without N and pH data since they were not measured in Lakes Koshkonong and Winnebago; and (B) all data, including N and pH, from Lakes Fish, Wingra, Monona, and Mendota. First, when all lakes were taken into consideration, Lake Koshkonong (*cos*2 = 0.88) had the largest influence on the first axis, which correlated to TP (*cos*2 = 0.96) and DRP (*cos*2 = 0.71) (Table S2a). Fish Lake (*cos*2 = 0.70) was anti-correlated to Lake Koshkonong on the first axis and was greatly influenced by Secchi depth (*cos*2 = 0.98). Lakes Mendota (*cos*2 = 0.79) and Monona (*cos*2 = 0.63) had the largest contribution to the second axis, which was positively correlated to maximum depth (*cos*2 = 0.5). Interestingly, Lake Winnebago (*cos*2 = 0.60 with first axis), which contained the highest “toxin” concentrations, was most influenced by surface area (*cos*2 = 0.57) and DRP (*cos*2 = 0.71), not chl-*a*, TP, or Secchi depth. Thus, it may be important to consider dissolved nutrient concentrations and/or fluxes (e.g., DRP), rather than just relying biomass indicators (e.g., chl-*a* or TP).

When all parameters were analyzed in Lakes Mendota, Monona, Wingra, and Fish, Lake Wingra had the greatest impact on the first axis (*cos*2 = 0.69) (Table S2b), and was anti-correlated to almost all variables (i.e., nutrients, size, and chl-*a*) except pH (*cos*2 = 0.03) and Secchi depth (*cos*2 = 0.63). Lake Mendota had the second most impact on the first axis (*cos*2 = 0.58) and was most influenced by lake depth (*cos*2 = 0.69), NH_4_^+^ (*cos*2 = 0.66), and N + N (*cos*2 = 0.58), while Lake Monona (*cos*2 = 0.56) was influence by TP (*cos*2 = 0.55). Again, in this analysis, chl-*a* (*cos*2 = 0.81 with the second axis) was most correlated with Fish Lake (*cos*2 = 0.76 with the second axis), which contained the second lowest “toxin” concentrations in this study.

## 3. Discussion

We surveyed six eutrophic Wisconsin lakes for 15 cyanobacterial metabolites, nine of which are known animal and human toxins. With the exception of Cpt1020, CYL, and SXT, all metabolites were detected at least once. To our surprise, we detected NOD in Lakes Wingra and Koshkonong. Though NOD has been more commonly associated with estuarine and brackish waters [29], it has also been documented in freshwater lakes [40,41,42], and in plant symbionts [43]. As in Wood et al. (2012), neither *Nodularia* nor any other source of NOD has been identified in these lakes. One explanation could be that historically phytoplankton counts have occurred in surface waters, and, thus, the presence of benthic cyanobacteria such as *Nodularia* has eluded us. Perhaps a more pressing question could be what are the drivers of NOD production in freshwater lakes. Lakes Koshkonong and Wingra are extremely shallow (~2–4 m maximum depth), and interestingly, surface water salinity in Lake Wingra has increased significantly (*p* < 0.01; R^2^ = 0.38) over the last two decades to a max of 170 mg·L^−1^, and to a lesser extent, has increased significantly in Lakes Mendota and Monona over the last 50 years, likely due to salting of roadways during the winter months [44]. Seeing as NOD has been shown to accumulate in benthic mats and crayfish [41], as well as plants [43], and is a potent hepatotoxin similar to MC, future studies regarding the distribution of NOD in freshwater lakes and lake macrofauna will be important for the protection of ecosystem services and public health.

We did not detect CYL, though *Cylindrospermopsis* has been previously identified in Lakes Mendota, Monona, and Wingra [45]. In addition, we did not detect SXT, which has been shown to be produced by *Cylindrospermopsis*, *Anabaena*, *Aphanizomenon*, *Lyngbya*, and others [46], all of which have been identified in Lakes Winnebago [47], Mendota, Monona, Wingra, and possibly Fish Lake (https://lter.limnology.wisc.edu/). In particular, *Aphanizomenon* has been shown to completely dominate Lakes Mendota [48] and Monona [45], but has never been shown to be toxic. Similarly, ATX and hATX were only detected once each in Lake Winnebago and Lake Monona, respectively. hATX has only been shown to be produced by *Anabaena flos-aquae* [49], but ATX has been shown to be produced by a number of cyanobacteria [46], including abundant cyanobacteria in our study lakes—*Aphanizomenon*, *Anabaena*, and *Microcystis.* The environmental triggers that promote STX, CYL, hATX, and ATX production are not known. However, the number of potential toxin producers in our study lakes and the lack of STX, CYL, hATX, and ATX detects suggests that either these cyanobacterial species lack the necessary genes for toxin production or the environmental conditions observed were not optimal for toxin biosynthesis.

MC was by far the most abundant “toxin” detected in this study. Of particular concern, MC was greater than 1 µg·L^−1^ in every sample collected from Lake Winnebago, which serves as a drinking water source for over 300,000 people. Similar levels were recorded in Lake Monona, which is a major recreational hub of the Madison Metropolitan Area. Interestingly, MCLR was detected in 100% of samples collected from Fish Lake but all concentrations were slightly below 1 µg·L^−1^. In a previous study in Lake Wingra, Beversdorf et al. (2015) [50] suggested that MC was constitutively expressed, possibly due to N stress [48], but low nutrient concentrations prevented high biomass, and thus MC concentrations, from ever being reached. Constant MC detects and low nutrient availability in Fish Lake would corroborate this observation. Similarly, Lake Monona had the lowest observed N:P ratios in our study lakes (Table 3), suggesting it may also be N stressed, but had higher nutrient concentrations than Fish Lake. Lake Monona had the second highest MC concentrations observed in this study. Comparatively, Lake Mendota had very similar physiochemical characteristics to Lake Monona, but with higher DIN concentrations and N:P ratios throughout the entire study (Table 3; see Figure S2 for temporal variability). As such, Lake Mendota had much lower MC concentrations than Lake Monona. While N was not measured in Lakes Winnebago and Koshkonong, DRP levels were highest in these lakes, suggesting they too could be N limited, at least for a portion of the year. Thus, the availability and proportion of dissolved nutrients may have a profound impact on cyanobacterial toxin concentrations.

While it was expected that MC would be the most prevalent toxin, Apts were the most frequently detected compounds and Cpts were also detected in approximately 50% of samples. This is extremely important because, although they are not considered toxins currently, Apts and Cpts may contribute to the overt toxicity of cyanobacterial blooms. For example, Chorus et al. (2001) [51] measured the toxicity of cyanobacterial extracts by bioassay and found that extracts eluted in 100% methanol were more toxic than those eluted in 75% methanol, and both were more toxic than what would be predicted by the amount of MC in the extract (measured by HPLC). Additionally, like MCs, AptF has been shown to inhibit protein phosphatases [25], albeit at a lower potency than MCs. The majority of Apts and Cpts have been shown to inhibit a variety of ubiquitous serine/threonine proteases such as kallikreins, trypsin, and chymotrypsin. As such, these compounds may have profound impacts not just on human health, but also on freshwater ecosystems as a whole. One study has shown that AptB and F lead to viral induced lysis of *Microcystis aeruginosa* cells [26]. While the Sedmak et al. (2008) study was performed in culture experiments and has yet to be demonstrated in lakes, the concentrations of Apt used were well within the range observed in our study. One difficulty in establishing the role of these peptides is that multiple species can synthesize multiple metabolites, and it has been reported that *Microcystis* itself produces Apt [52]. Similarly, it appears *Microcystis* is the major producer of Cpt in freshwater lakes [5]. While Cpt has been classified as a potential neurotoxin following zebra fish toxicity assays [20] and is toxic to at least one freshwater crustacean [21], no studies have demonstrated its possible toxicity to mammals, including humans. Ultimately, it is possible that the plethora of these metabolites provides these species with some sort of functional redundancy. While some culture studies have shown that toxic cyanobacterial species can outcompete nontoxic species, field-based studies have shown that cyanobacterial communities are almost always comprised of both nontoxic and toxic species and that their abundances are highly variable.

A goal of this study was to determine the distribution of these compounds/toxins in six similar eutrophic lakes that have all experienced cyanobacterial blooms. We anticipated that although watershed and biogeochemical similarities exist between these lakes, including cyanobacterial community composition [45,48,53], differences would exist in their metabolite profiles. Surface water temperature and pH were virtually identical in all six lakes, and no significant differences existed between chl-*a* concentrations. Thus, the major differences between lakes, as measured in this survey, were between lake size and N and P concentrations. Lakes Winnebago and Koshkonong, which had the highest cyanobacterial metabolite and P concentrations, were the largest lakes by surface area. Lake Monona, the next most “toxic” lake, also had comparatively high P concentrations, but also had lower dissolved N concentrations compared to Lake Mendota (the next most toxic lake). Lake Wingra and Fish Lake had the lowest N and P concentrations and the lowest metabolite concentrations. Despite differences between lakes, many of the compounds measured correlated to each other within lakes. Thus, it is possible that: (A) one species was producing the majority of the compounds observed; and/or (B) the environmental trigger(s) for each compound is(are) similar. Future studies eliciting the cellular function of these compounds are needed. Additionally, with recent freshwater regulations being employed for MC and CYL, the health impacts of other cyanobacterial metabolites individually and in mixtures need to be deciphered.

## 4. Conclusions

- We measured 15 cyanobacterial compounds, nine of which are known human toxins, in six eutrophic lakes.
- Anabaenopeptins were the most frequently detected compounds, followed by microcystins and cyanopeptolins.
- In two highly populated lakes, one of which is used as a drinking water source, total microcystin concentrations were above 1 µg·L^−1^ in all samples collected.
- Nodularin, a more common marine toxin, was detected in the two shallowest lakes.
- Lake size and dissolved nitrogen and phosphorus concentrations (not TN and TP) were the most probable variables driving metabolite profiles.
- In order to determine the impact of these metabolites on ecosystem function and public health and manage them appropriately, similar studies are needed to elicit the eco-physiological role of these toxins.

## 5. Materials and Methods

### 5.1. Study Sites

From 13 April 13 to 10 October 2012, surface water samples (0–0.5 m) were collected from six eutrophic Wisconsin lakes: Fish (*n* = 10), Wingra (*n* = 10), Monona (*n* = 10), Mendota (*n* = 9), Koshkonong (*n* = 10), and Winnebago (*n* = 10) (Figure 1). All lakes have been categorized as meso/eutrophic, eutrophic, or hyper-eutrophic by the Wisconsin Department of Natural Resources using chlorophyll-*a* (chl-*a*) and total phosphorus (TP) concentrations and trophic index (Table 1). Lakes ranged in size by surface area, depth, and volume with Fish Lake being the smallest by area (0.87 km^2^) and Lake Winnebago being by far the largest (534 km^2^) (Table 1). Lake Mendota was the deepest lake at 25.3 m and Lake Koshkonong, despite being 43 km^2^, had a max depth of only 2.1 m. By volume, Lake Winnebago was largest (1709 × 10^6^ m^3^) and Lake Wingra was the smallest (3 × 10^6^ m^3^).

Lakes Koshkonong, Monona, Mendota, Wingra, and Winnebago are considered drainage lakes (i.e., have both an inlet and outlet) while Fish Lake is considered a seepage lake (i.e., has no inlet or outlet and the main source of water is precipitation and runoff). Lake Winnebago is primarily fed from the west by the Lower Fox River Basin through Lakes Poygan, Winnecone, and Butt des Morts and exits to the north via the Upper Fox River Basin eventually flowing into Green Bay, Lake Michigan. Lakes Mendota and Monona are fed primarily by the Yahara River and Lake Koshkonong is fed by the Rock River. Even though Lake Wingra is considered a drainage lake, it lacks a major river inflow and is instead fed by precipitation, surface runoff, and ground water to varying degrees throughout the year and from year to year. Its major outlet is Wingra Creek, which empties into Monona Bay of Lake Monona.

### 5.2. Sample Collection and Preservation

Surface water samples (0–0.5 m) were collected from the deepest area of each lake over the course of the 2012 open-water season (*n* = 59). All samples were collected in amber glass bottles, immediately placed on ice, and transported to the lab where half the water sample was filtered through 0.2 µm, 47 mm diameter SUPOR membrane filter (Pall Life Sciences, Ann Arbor, MI, USA) for chl-*a* and dissolved reactive P (DRP) analysis. Both unfiltered and filtered water samples were then stored at −20 °C until further analysis.

### 5.3. Chemicals

Fifteen cyanobacterial metabolites were analyzed as part of this study, including six hepatotoxins, six bioactive cyanopeptides, and three neurotoxins. For the hepatotoxins, certified reference material for microcystin-LR (MCLR) was purchased from the National Research Council of Canada Biotoxins program (Halifax, NS, Canada). Nodularin (NOD) (purity >94%), MCLA (>95%), MCYR (>90%), and MCRR (>90%) were purchased from Sigma-Aldrich (Milwaukee, WI, USA), and cylindrospermopsin (CYL) (>95%) was purchased from Abraxis (Warminster, PA, USA). For the peptides, anabaenopeptin B (Apt B) (>95%) and AptF (>95%), cyanopeptolin 1007 (Cpt1007) (>95%), Cpt 1021 (>95%), and Cpt 1040 (>95%), and microginin 690 (Mgn690) (>95%) were purchased from MARBIONC (Wilmington, NC, USA). Additionally, the neurotoxins anatoxin-a (ATX), homoanatoxin-a (hATX), and saxitoxin (SXT) were also targeted in this study. ATX fumarate (96%) was purchased from Tocris Bioscience (Minneapolis, MN, USA) as a racemic mixture, hATX (>95%) was purchased from Abraxis, and SXT was purchased from the National Research Council of Canada Biotoxins program (Halifax, NS, Canada).

### 5.4. Chemical Extraction and Analysis

Samples were thawed overnight at 4 °C and then to completion in a 50 °C water bath. For the extraction of peptides, including MCs, NOD, Apts, Cpts, and Mgn690, precisely 10 mL of sample was transferred to a conical tube and frozen to completion at −80 °C for 30 min. Frozen samples were lyophilized (Labconco FreeZone 6L, Kansas City, MO, USA) for 48 hours and the dried mass was resuspended in 1 mL of water containing 0.1% formic acid. The suspension was then subject to three freeze/thaw cycles at -80 °C and 50 °C, respectively. After the final thaw, 2 mL of 100% methanol (MeOH) was added for a final extraction concentration of 67% MeOH. The extract was sonicated for 10 min at 45 °C (SharperTEK Stamina XP Heated Ultrasonic Cleaner, Pontiac, MI, USA), then centrifuged for 15 min at 5000× *g*, and the supernatants were stored at −20 °C in amber vials until analysis. The extraction efficiencies were tested in ten percent of samples by adding a known amount of each peptide to water samples prior to extraction.

For ATX, hATX, and CYL, precisely 1 mL of sample was transferred to a 1.5 mL microcentrifuge tube and then spiked with 10 µL of 0.5 mg·L^−1^ ^13^C_6_-phenylalanine (>99%, Cambridge Isotopes, Tewksbury, MA, USA) plus formic acid (final concentration 0.1%). The ^13^C_6_-phenylalanine served as a surrogate standard to monitor analyte recovery and to differentiate between ATX and phenylalanine given their similar retention times, same molecular weight, and identical product ion spectrums. Sample extracts were then subject to three freeze/thaw cycles, sonicated in a water bath, and then centrifuged, and the supernatants were stored in amber vials until analysis as described above.

All toxins and peptides were analyzed by liquid chromatography tandem (MS/MS) mass spectrometry on an ABSciex 4000 QTrap Mass Spectrometer (Framingham, MA, USA) equipped with a TurboV™ electrospray ion source and a Shimadzu HPLC Model 20A (Kyoto, Japan). Chromatographic separation of peptides was achieved on a reverse phase C18 column (Luna®, 3 µm, 100 Å, LC Column 150 × 3 mm, Phenomenex, Torrance, CA, USA) using mobile phases (A) HPLC water with 0.1% formic acid and 5 mM ammonium acetate; and (B) 95% acetonitrile with 0.1% formic acid and 5 mM ammonium acetate. Gradient elution was used for the C18 method consisting of 30% B for 3 min., increasing on a linear gradient to 95% B at 9 min., held at 95% B until 15 min., and returned to 30% B until 20 min. SXT, ATX, hATX, ^13^C_6_-phenylalanine, and CYL were separated by hydrophilic interaction chromatography (HILIC) (SeQuant®, 5 μm, 150 × 2.1 mm I.D., EMD Millipore Corporation, Billerica, MA, USA) with mobile phases of (A) HPLC water with 60 mM formic acid and (B) 100% acetonitrile with 60 mM formic acid. Isocratic elution (60% B) was used for the HILIC method.

Optimal mass spectrometry conditions were optimized for each compound separately. A 1000 µg·L^−1^ standard of each compound in 50% methanol/0.1% formic acid was syringe infused into the mass spectrometer at 10 µL·min^−1^ and product ion spectra recorded (Figures S3–S5). Optimal compound specific parameters including collision energy, entrance potential, and declustering potential (Table S3) were determined using Analyst 1.5 (Sciex, Concord, ON, Canada). Flow injection analysis was used to select optimal ion source gas flow rates, ionization energy and temperature (Table S4). Two daughter ions were chosen for each compound based on maximum signal-to-noise ratios in chromatographic runs and one was chosen for quantification (Figures S6–S8). The quantitation limit for all compounds was 0.05 µg·L^−1^ for all compounds and this was also set as the detection limit since lower standard concentrations rarely produced 3:1 signal-to-noise ratios for most compounds.

Product ion spectra of ATX and phenylalanine are identical [54]. Similarly, product ion spectra of AptB/AptF, and Cpt1020/Cpt1007 are also nearly identical (Figure S4) [51]. Therefore, LC methods were employed to adequately separate these compounds chromatographically. Peaks of ATX and ^13^C_6_-phenylalanine were separated by >15 s using HILIC chromatography (Figure S8). Likewise, AptB and AptF were separated by 1.1 min and Cpt1020 was separated from Cpt1007 by 0.6 min using reverse phase (C18) chromatography (Figure S7). To ensure adequate separation between ATX and phenylalanine across all samples, ^13^C_6_-phenylalanine was monitored in every HILIC run.

### 5.5. Ancillary Analytical Data and Measurements

Lakes Mendota, Monona, Wingra, and Fish are sampled biweekly as part of the North Temperate Lakes-Long Term Ecological Research Program (NTL-LTER). Toxin and peptide samples were collected and analyzed as described above. All other protocols are available online (http://lter.limnology.wisc.edu) and have been previously published [48]. Briefly, for Lakes Fish, Mendota, Monona, and Wingra, ammonium was measured spectrophotometrically at 660 nm after conversion to indophenol. Nitrate and nitrite (N + N) were simultaneously measured spectrophotometrically at 520 nm following cadmium reduction. DRP was measured spectrophotometrically at 880 nm following conversion to the phosphomolybdenum complex. TP and total N (TN) were first digested by addition of sodium hydroxide-potassium persulfate and then autoclaving; TP and TN were then measured as DRP and N + N, respectively. N and P measurements were conducted on an Astoria-Pacific Astoria II segmented flow autoanalyzer. Chl-*a* was extracted with 100% methanol and measured fluorometrically (Turner Designs TD-700, San Jose, CA, USA).

For Lake Winnebago and Lake Koshkonong, TP data were received from the Wisconsin Department of Natural Resources, which regularly monitors water quality at these locations, and was analyzed following EPA Method 365.1. Data from these lakes are also available online (http://dnr.wi.gov/lakes). DRP and chl-*a* were measured in our lab by the ascorbic acid-molybdenum blue method 4500-P E [55] and spectrophotometrically after extraction with acetone [56] using coefficients from Porra et al. (1989) [57], respectively.

### 5.6. Statistical Analysis

All analyses were performed in Matlab [58] and R Project for Statistical Computing [59] (see below for functions in italics). Within lake samples were tested for normality using the Lilliefors Test (*lillietest*), which uses a post-hoc Monte Carlo simulation to determine if the null hypothesis can be rejected (i.e., data are not normally distributed). Based on those results, we compared in-lake metabolites using a Spearman (*corr*) rank correlation. Relationships between lakes were tested using the Kruskal–Wallis test (*kruskalwallis*), which returns the probability that the data come from the same distribution, followed by a post-hoc analysis using the Dunn–Sidak method (*multcompare*). All parameters were considered significant at *p* < 0.05. To test temporal and spatial analyses of lake metabolite profiles, principal component analyses (PCA) were performed in *R* Statistical Packages *FactoMineR* and *factoextra* using the *PCA* function. All data were first log transformed and an analysis of similarity (*anosim*) was performed using Euclidean distance and 999 random permutations to establish significance.

## Figures and Tables

**Table 1:** General lake characteristics for six lakes surveyed as a part of this study.

Lake	Trophic State *	Lake Type	Majority Land Use	Area (km^2^)	Max Depth (m)	Volume (10^6^ m^3^) **	Secchi (m) ***
Fish	Meso/eutrophic	Seepage	Ag	0.87	18.9	8.2	2.8
Wingra	Eutrophic	Drainage	Ur	1.40	4.3	3.0	2.2
Monona	Eutrophic	Drainage	Ur/Ag	13.2	22.5	149	1.2
Mendota	Eutrophic	Drainage	Ur/Ag	39.4	25.3	498	1.7
Koshkonong	Hyper/eutrophic	Drainage	Ag	42.9	2.1	45.0	0.3
Winnebago	Hyper/eutrophic	Drainage	Ur/Ag	534	6.4	1709	0.6

* Determined by the Wisconsin DNR based on the trophic state index (TSI) using chlorophyll, total phosphorus, and/or Secchi depth; ** Assuming mean depth is half the max depth; *** Average summer Secchi depth; Ag = agricultural; Ur = Urban.

**Table 2:** Mean, median, and max concentrations, as well as the variance, of metabolites observed in this study. For all lakes, *n* = 10, with the exception of Lake Mendota where *n* = 9. BDL = below detection limit of 0.01 µg·L^−1^.

**Lake**	**Function**	**MCLR**	**MCYR**	**MCLA**	**MCRR**	**Total MC**	**Apt B**	**AptF**	**Total Apt**	**NOD**
Fish	Mean	0.48	BDL	0.03	BDL	0.51	0.45	0.01	0.46	BDL
	Median	0.44	BDL	0.03	BDL	0.47	0.28	BDL	0.28	BDL
	Variance	0.06	0.00	0.00	0.00	0.06	0.26	0.00	0.26	0.00
	Max	0.87	BDL	0.07	BDL	0.94	1.78	0.08	1.86	BDL
Koshkonong	Mean	0.61	0.01	BDL	0.04	0.65	5.97	0.59	6.56	0.06
	Median	0.26	BDL	BDL	0.02	0.28	4.13	0.15	4.28	BDL
	Variance	1.03	0.00	0.00	0.00	1.03	57.54	1.04	58.58	0.02
	Max	3.32	0.03	0.03	0.13	3.51	24.05	3.40	27.45	0.35
Monona	Mean	4.83	BDL	0.35	0.01	5.20	0.73	0.07	0.80	BDL
	Median	3.22	BDL	0.17	0.01	3.40	0.36	0.05	0.41	BDL
	Variance	21.35	0.00	0.18	0.00	21.54	1.05	0.01	1.06	0.00
	Max	12.40	0.03	1.11	0.04	13.58	3.41	0.24	3.65	BDL
Mendota	Mean	0.38	BDL	0.19	BDL	0.58	0.21	0.01	0.22	BDL
	Median	0.34	BDL	0.08	BDL	0.42	0.08	BDL	0.08	BDL
	Variance	0.18	0.00	0.05	0.00	0.23	0.07	0.00	0.07	0.00
	Max	1.11	0.03	0.60	0.02	1.76	0.63	0.03	0.66	BDL
Wingra	Mean	0.10	BDL	0.01	BDL	0.11	0.09	0.02	0.11	0.06
	Median	BDL	BDL	BDL	BDL	0.01	0.06	0.02	0.08	BDL
	Variance	0.02	0.00	0.00	0.00	0.02	0.01	0.00	0.01	0.03
	Max	0.33	BDL	0.02	0.02	0.37	0.25	0.06	0.31	0.54
Winnebago	Mean	12.21	0.19	0.30	1.18	13.87	1.64	0.66	2.30	BDL
	Median	6.37	0.07	0.07	0.56	7.07	0.97	0.52	1.49	BDL
	Variance	142.79	0.05	0.14	1.36	144.34	5.05	0.28	5.33	0.00
	Max	38.25	0.53	0.96	3.67	43.41	7.64	1.73	9.37	BDL
**Lake**	**Function**	**Cpt1007**	**Cpt1041**	**Cpt1020**	**Total Cpt**	**Mgn690**	**ATX**	**hATX**	**STX**	**CYL**
Fish	Mean	0.04	0.06	BDL	0.11	0.01	BDL	BDL	BDL	BDL
	Median	0.03	0.06	BDL	0.08	BDL	BDL	BDL	BDL	BDL
	Variance	0.00	0.00	0.00	0.01	0.00	0.00	0.00	0.00	0.00
	Max	0.14	0.14	BDL	0.28	0.04	BDL	BDL	BDL	BDL
Koshkonong	Mean	0.19	BDL	BDL	0.19	0.23	BDL	BDL	BDL	BDL
	Median	BDL	BDL	BDL	BDL	BDL	BDL	BDL	BDL	BDL
	Variance	0.37	0.00	0.00	0.37	0.48	0.00	0.00	0.00	0.00
	Max	1.92	BDL	BDL	1.92	2.21	BDL	BDL	BDL	BDL
Monona	Mean	0.11	0.55	BDL	0.67	0.05	BDL	0.51	BDL	BDL
	Median	0.11	0.54	BDL	0.65	0.01	BDL	BDL	BDL	BDL
	Variance	0.01	0.22	0.00	0.23	0.01	0.00	2.60	0.00	0.00
	Max	0.28	1.17	BDL	1.45	0.35	BDL	5.10	BDL	BDL
Mendota	Mean	0.04	0.26	BDL	0.30	BDL	BDL	BDL	BDL	BDL
	Median	BDL	0.36	BDL	0.36	BDL	BDL	BDL	BDL	BDL
	Variance	0.00	0.06	0.00	0.07	0.00	0.00	0.00	0.00	0.00
	Max	0.13	0.65	BDL	0.78	0.01	BDL	BDL	BDL	BDL
Wingra	Mean	BDL	BDL	BDL	BDL	0.01	BDL	BDL	BDL	BDL
	Median	BDL	BDL	BDL	BDL	BDL	BDL	BDL	BDL	BDL
	Variance	0.00	0.00	0.00	0.00	0.00	0.00	0.00	0.00	0.00
	Max	BDL	BDL	BDL	0.01	0.02	BDL	BDL	BDL	BDL
Winnebago	Mean	1.23	1.82	BDL	3.05	0.02	0.03	BDL	BDL	BDL
	Median	0.75	1.32	BDL	2.07	0.02	BDL	BDL	BDL	BDL
	Variance	3.35	4.47	0.00	7.83	0.00	0.01	0.00	0.00	0.00
	Max	6.38	7.54	BDL	13.92	0.07	0.30	BDL	BDL	BDL

**Table 3:** Chlorophyll-*a* and nutrient concentrations, nutrient ratios, and pH for all lakes surveyed in this study.

Lake	Function	Chl-*a*	TP	DRP	TN	N + N	NH_4_^+^	TN:TP	DIN:DRP	pH
Fish	Mean	14.7	14.6	3.60	798	16.7	85.7	9900	970	8.5
(*n* = 13)	Median	13.8	15.0	1.00	716	5.00	12.0	57.3	174	8.7
	Max	28.2	45.0	14.5	1170	62.0	504.0	72000	6900	9.1
Koshkonong	Mean	27.2	501	61.4	ND	ND	ND	ND	ND	ND
(*n* = 10 *)	Median	17.8	36.0	9.00	ND	ND	ND	ND	ND	ND
	Max	72.1	507	233	ND	ND	ND	ND	ND	ND
Monona	Mean	10.8	62.4	17.0	970	54.6	89.8	17.9	820	8.6
(*n* = 14)	Median	9.85	45.5	2.50	911	0.01	25.5	17.6	9.00	8.8
	Max	37.7	230	78.9	2937	229	314	32.1	630	8.9
Mendota	Mean	9.49	44.9	16.2	750	267	126	20.6	1195	8.5
(*n* = 17)	Median	7.75	46.5	12.0	786	135	77.0	17.5	16.7	8.6
	Max	26.7	112	49.0	1210	627	436	40.0	18800	8.9
Wingra	Mean	8.15	28.9	0.53	728	27.9	17.9	26.4	3800	8.6
(*n* = 15)	Median	5.85	29.0	0.00	720	0.00	0.00	24.0	1600	8.4
	Max	18.9	44.0	3.00	923	211	88.0	38.7	21000	9.2
Winnebago	Mean	11.0	149	30.4	ND	ND	ND	ND	ND	ND
(*n* = 10 *)	Median	9.80	139	27.5	ND	ND	ND	ND	ND	ND
	Max	16.9	195	78.0	ND	ND	ND	ND	ND	ND

ND = no data; * For Lakes Koshkonong and Winnebago, *n* = 3 for total phosphorus only. Chl-*a* = chlorophyll-*a*, TP = total phosphorus, DRP = dissolved reactive phosphorus, TN = total nitrogen, N + N = nitrate + nitrite, NH_4_^+^ = ammonium, DIN = dissolved inorganic nitrogen.

## Supplementary Materials

The following are available online at www.mdpi.com/2072-6651/9/2/62/s1, Figure S1: Principal component analysis and physiochemical characteristics of study lakes, Figure S2: Nitrogen and phosphorus ratios for Lakes Mendota and Monona, Figure S3: Product ion spectra for microcystins and nodularin, Figure S4: Product ion spectra for anabaenopeptins, cyanopeptolins, and microginin, Figure S5: Product ion spectra for cylindrospermopsin, anatoxin-a, homoanatoxin-a, ^13^C_6_-phenylalanine, and saxitoxin, Figure S6: Chromatograms produced from a 10 µg·L^−1^ standard of microcystin and nodularin, Figure S7: Chromatograms produced from a 10 µg·L^−1^ standard of cyanopeptolin, anabaenopeptin, and microginin, Figure S8: Chromatograms produced from a 10 µg·L^−1^ standard of anatoxin-a, ^13^C_6_-phenylalanine, cylindrospermopsin, homoanatoxin-a, and saxitoxin, Table S1: Contribution of metabolite classes to principal component analysis axes, Table S2: Contribution of physiochemical characteristics to principal component analysis axes, Table S3: Optimized tandem mass spectrometer settings for target analytes, Table S4: Ion source turbo spray settings.
