# Supplementary material for: Variable Cyanobacterial Toxin and Metabolite Profiles across Six Eutrophic Lakes of Differing Physiochemical Characteristics
Source: Toxins (Basel). 2017 Feb 10;9(2):62. doi: 10.3390/toxins9020062 (PMC5331441; doi:10.3390/toxins9020062)
Supplement: Supplementary file 1 [file toxins-09-00062-s001.pdf]

Supplementary Materials: Variable Cyanobacterial Toxin and Metabolite Profiles across Six Eutrophic Lakes of Differing Physiochemical Characteristics

Lucas J. Beversdorf, Chelsea A. Weirich, Sarah L. Bartlett and Todd. R. Miller

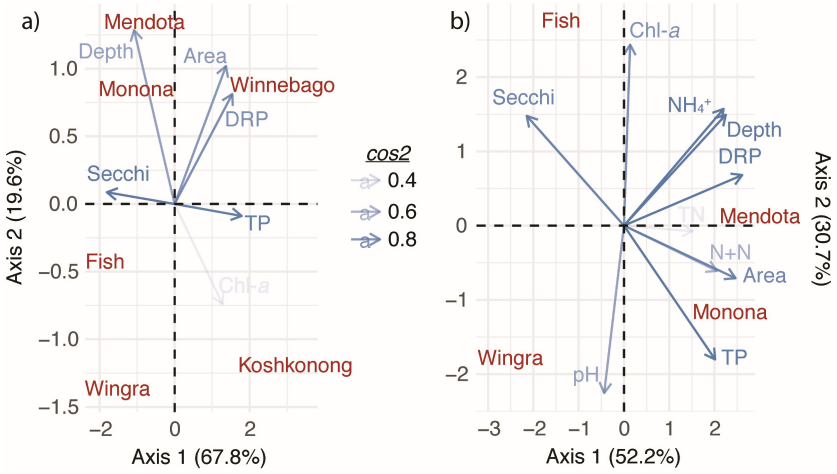

Figure S1. Principal component analysis of (**a**) all six lakes in this study and physiochemical characteristics with the exception of nitrogen and pH observations, since they were not measured in Lakes Winnebago and Koshkonong and (**b**) all available physiochemical characteristics that were measured in Lakes Mendota, Monona, Wingra, and Fish. Lakes are separated by Euclidean distance where the closer samples are to each other, the more similar their lake characteristics are. Lakes were significantly different based on analysis of similarity (ANOSIM; $p < 0.05$). Arrows point in the direction of samples with higher correlations and the length of each arrow represents the magnitude of that correlation squared (*cos*2). TN = total nitrogen; N + N = nitrate + nitrite; NH_4^+ = ammonium; TP = total phosphorus; DRP = dissolved reactive phosphorus; Chl-*a* = chlorophyll-*a*; Depth = maximum depth of lake.

Table S1. Contribution of each lake and cyanobacterial metabolite to the first and second axes of the principal component analysis performed.

Lake	Axis 1	Axis 2
Winnebago	6.15	0.40
Wingra	5.42	0.45
Monona	3.53	1.46
Mendota	2.66	1.68
Fish	2.35	0.87
Koshkonong	1.82	3.08
**Variable**	**Axis 1**	**Axis 2**
Cpt1007	0.71	0.04
MCRR	0.69	0.04
MCLR	0.61	0.09
MCYR	0.55	0.02
Cpt1041	0.54	0.18
AptF	0.52	0.28
MCLA	0.35	0.22
AptB	0.34	0.21
Mgn690	0.21	0.10
ATX	0.06	0.01
NOD	0.03	0.36
hATX	0.00	0.14

Table S2. (**a**) Contribution of each lake and physiochemical characteristic to the first and second axes of the principal component analysis performed. (**b**) Contribution of each lake and physiochemical characteristic to the first and second axes of the principal component analysis performed.

	(**a**)	
**Lake**	**Axis 1**	**Axis 2**
Koshkonong	0.88	0.10
Fish	0.70	0.04
Winnebago	0.60	0.19
Wingra	0.56	0.25
Mendota	0.21	0.79
Monona	0.10	0.63
**Variable**	**Axis 1**	**Axis 2**
Secchi	0.98	0.00
TP	0.96	0.00
DRP	0.71	0.20
Area	0.57	0.31
Chl-*a*	0.49	0.16
Depth	0.35	0.50
	(**b**)	
**Lake**	**Axis 1**	**Axis 2**
Wingra	0.69	0.30
Mendota	0.58	0.00
Monona	0.56	0.11
Fish	0.22	0.76
**Variable**	**Axis 1**	**Axis 2**
DRP	0.93	0.06
Area	0.83	0.07
Depth	0.69	0.30
NH^{4}_{+}	0.66	0.34
Secchi	0.63	0.30
N + N	0.58	0.05
TP	0.55	0.44
TN	0.32	0.00
pH	0.03	0.69
Chl-*a*	0.00	0.81

Table S3. Optimized MS/MS settings for target analytes.

Analyte	Formula	Parent	Daughter	RT	DP	EP	CE	%Recovery	CXP
MCLR	$C_{49}H_{74}N_{10}O_{12}$	995.6	135.3	8.4	126	10	115	78	26
		995.6	127.1	8.4	126	10	115		26
MCYR	$C_{52}H_{72}N_{10}O_{13}$	1045.6	135.3	8.3	141	10	107	68	8
		1045.6	127.1	8.3	141	10	123		8
MCLA	$C_{46}H_{67}N_{7}O_{12}$	910.6	776.4	10.2	106	10	27	80	8
		910.6	135.2	10.2	106	10	87		8
MCRR	$C_{49}H_{75}N_{13}O_{12}$	520.0	70.1	7.5	56	10	129	76	6
		520.0	135.1	7.5	81	10	43		8
AptB	$C_{41}H_{60}N_{10}O_{9}$	837.5	201.4	4.7	106	10	57	85	14
		837.5	70	4.7	106	10	129		10
AptF	$C_{42}H_{62}N_{10}O_{9}$	851.8	201	5.8	121	10	53	73	12
		851.8	175.1	5.8	121	10	53		12
Cpt1007	$C_{49}H_{70}N_{10}O_{13}$	1007.5	989.6	8	131	10	51	84	32
		1007.5	776.3	8	131	10	59		22
Cpt1041	$C_{49}H_{69}ClN_{10}O_{13}$	1042.5	1024.5	8.3	131	10	51	78	28
		1042.5	184.2	8.3	131	10	109		8
Cpt1020	$C_{50}H_{72}N_{10}O_{13}$	1021.6	989.6	8.6	131	10	57	64	32
		1021.6	776.4	8.6	131	10	63		22
Mgn690	$C_{34}H_{50}N_{4}O_{9}S$	691.4	510.2	5.3	96	10	31	76	16
		691.4	343.1	5.3	96	10	37		10
$^{13}C_6$-Phe	$C_{9}H_{11}NO_{2}$	172.1	126.1	3.1	41	10	19	99 ± 23	8
		172.1	109.2	3.1	41	10	39		6
ATX	$C_{10}H_{15}NO$	166.1	149.3	1.8	46	10	21	-	10
		166.1	131.3	1.8	46	10	25		8
		166.1	107.2	1.8	46	10	25		6
hAtx	$C_{11}H_{17}NO$	180.1	163.3	1.6	51	10	19	-	10
		180.1	145.3	1.6	51	10	23		10
CYL	$C_{15}H_{21}N_{5}O_{7}S$	416.2	194.0	1.6	71	10	49	85	10
		416.2	336.2	1.6	71	10	31		10
SXT	$C_{11}H_{17}NO$	300.2	282.1	8.7	101	10	25	-	22
		300.2	204.0	8.7	101	10	33		14
NOD	$C_{41}H_{60}N_{8}O_{10}$	826.5	103.2	8.0	116	10	83	-	8
		826.5	135.3	8.0	116	10	129		16

RT = retention time, DP = declustering potential, EP = entrance potential, CE = collision energy, CXP = collision cell exit potential.

Table S4. Ion source turbo spray settings.

Parameter	Setting
Curtain Gas (psi)	20
Collision Gas (psi)	High
Ion Spray Voltage (psi)	5500
Temperature (C)	700
Ion Source Gas 1 (psi)	70
Ion Source Gas 2 (psi)	70

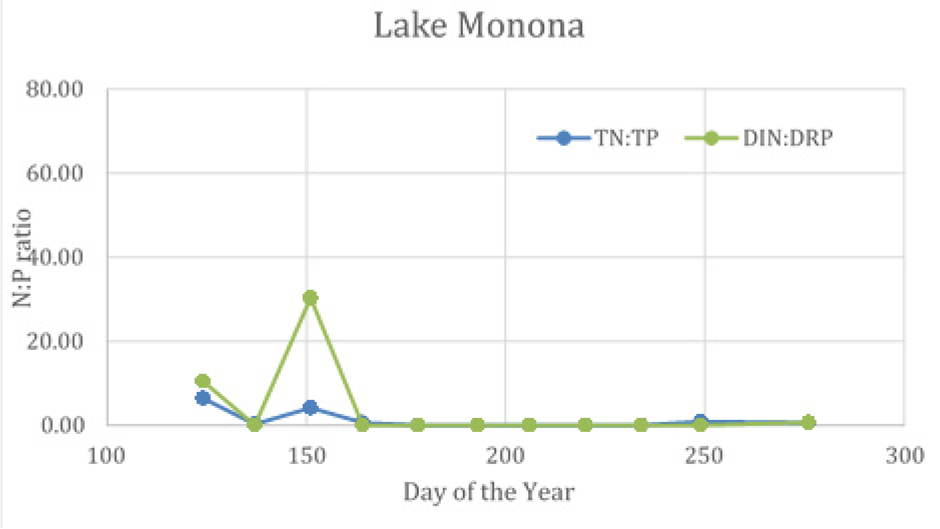

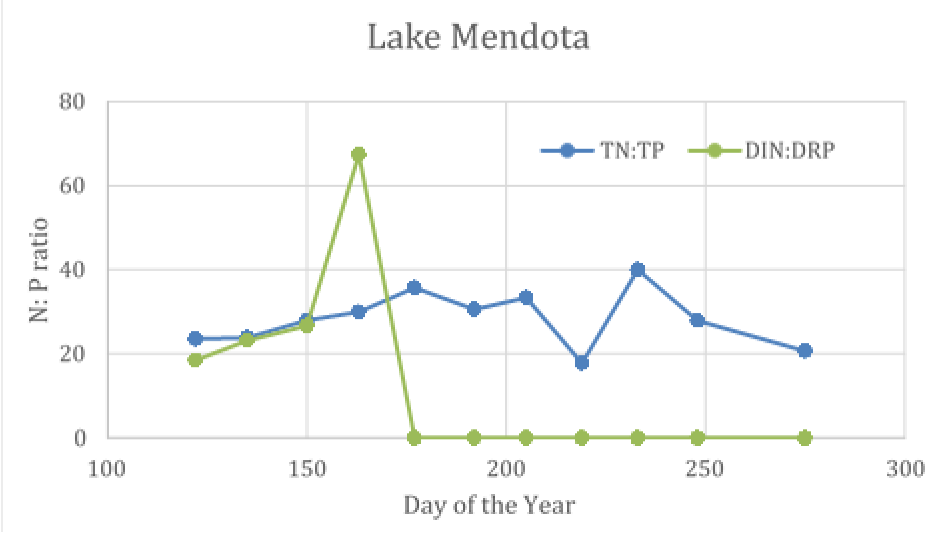

Figure S2. Nitrogen (N) to phosphorus ratios (P) for Lakes Monona and Mendota. TN and DIN = total and dissolved inorganic nitrogen; TP and DRP = total and dissolved reactive phosphorus.

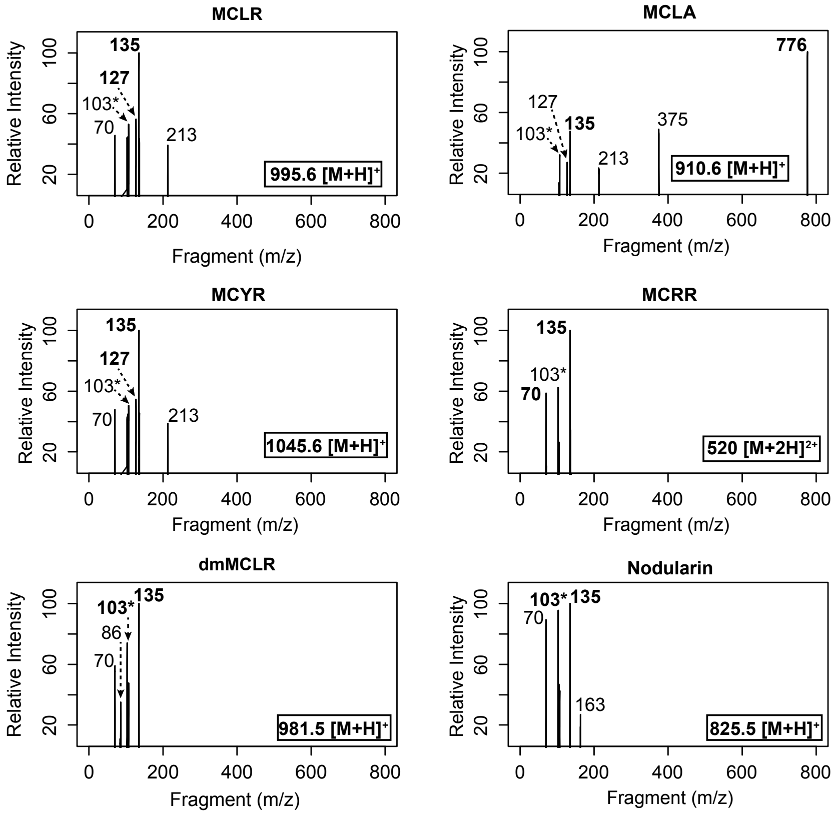

Figure S3. Product ion spectra for microcystins and nodularin. The peak near 103 *m*/*z* for microcystins is composed of three ions of approximately 103, 104, 105, and/or 107 *m*/*z*.

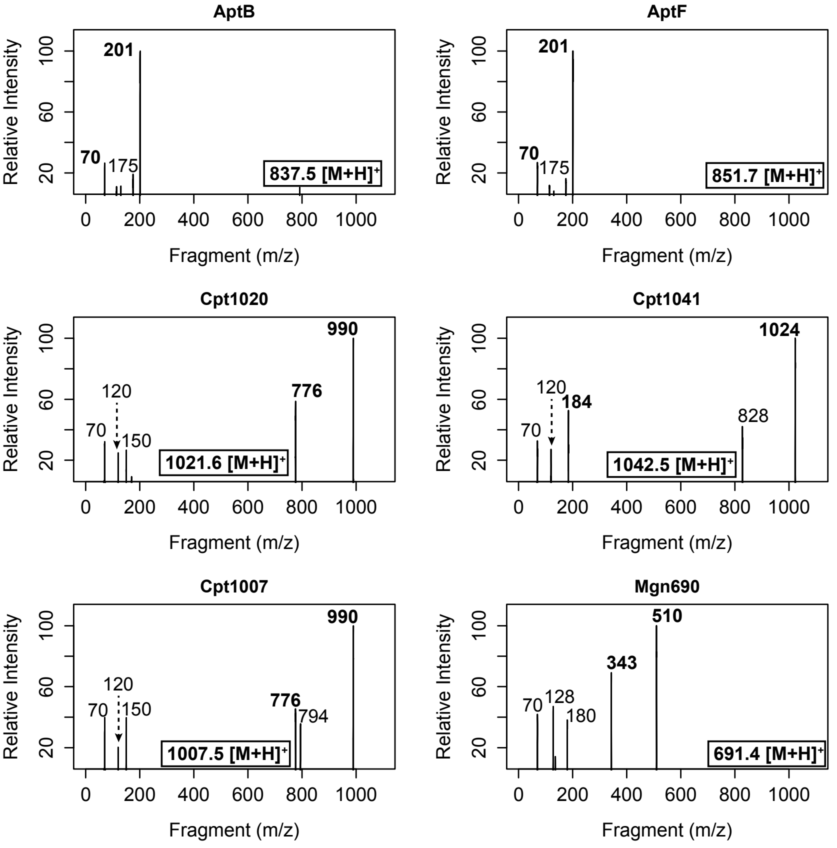

Figure S4. Product ion spectra for anabaenopeptins (Apt), cyanopeptolins (Cpt) and microginin-690 (Mgn690).

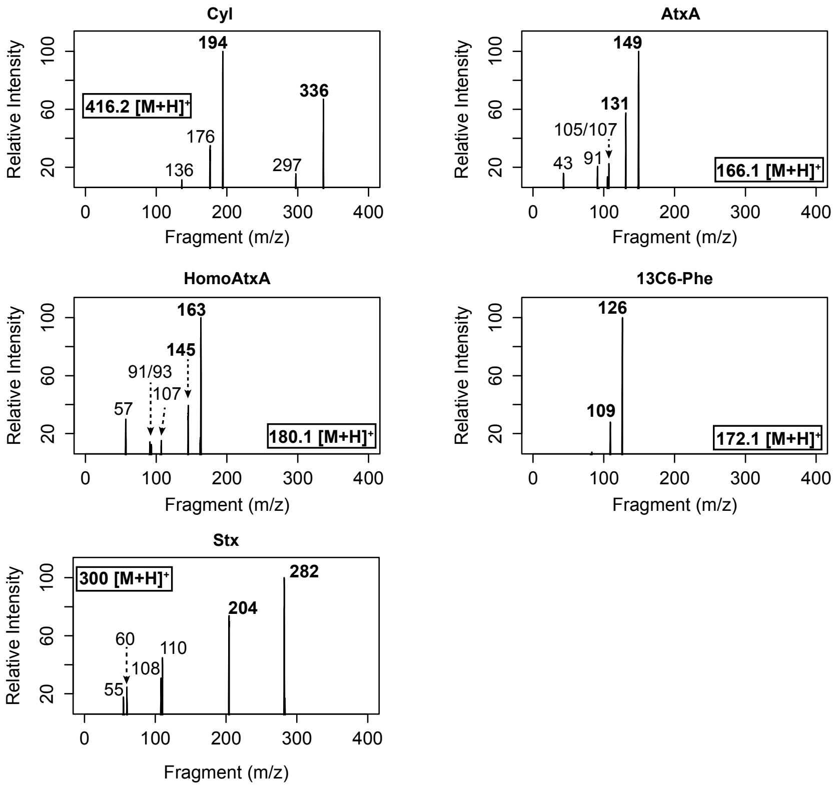

Figure S5. Product ion spectra for cylindrospermopsin (Cyl), anatoxin-a (AtxA), homoanatoxin-a (HomeAtxA), $^{13}C_6$-phenylalanine ($^{13}C_6$-Phe), and saxitoxin (Stx).

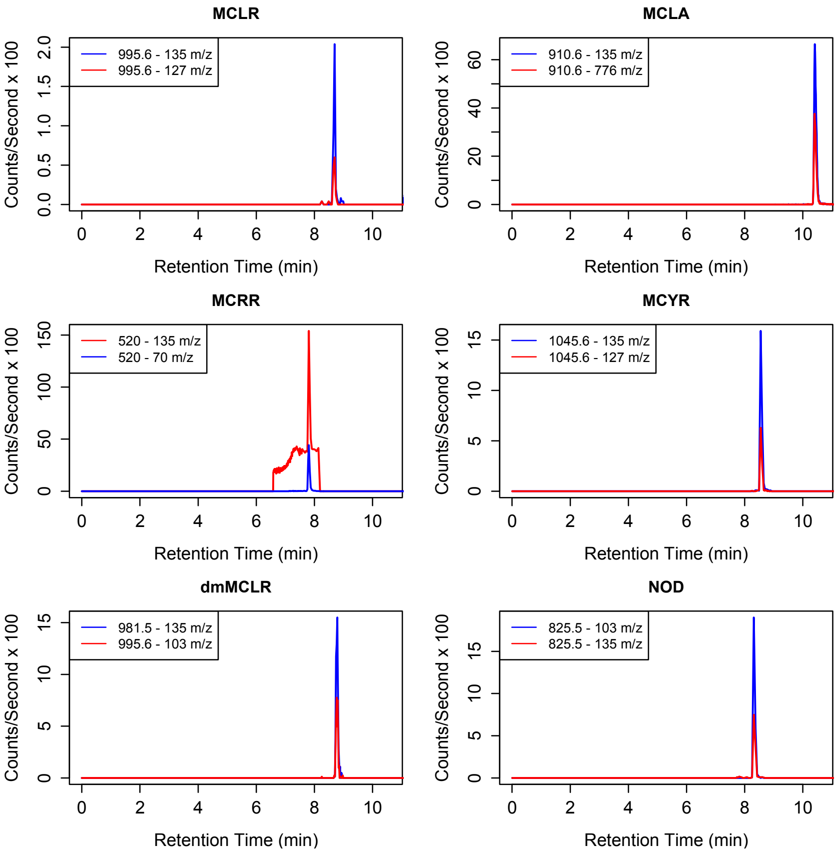

Figure S6. Chromatograms produced from a 10 μg·L^{-1} standard of microcystins and nodularin. Transition ions in blue were chosen for quantitation, and red transitions are confirmatory ions. MCLR = microcystin-LR, MCLA = microcystin-LA, MCRR = microcystin-RR, MCYR = microcystin-YR, dmMCLR = desmethyl microcystin- LR, NOD = nodularin.

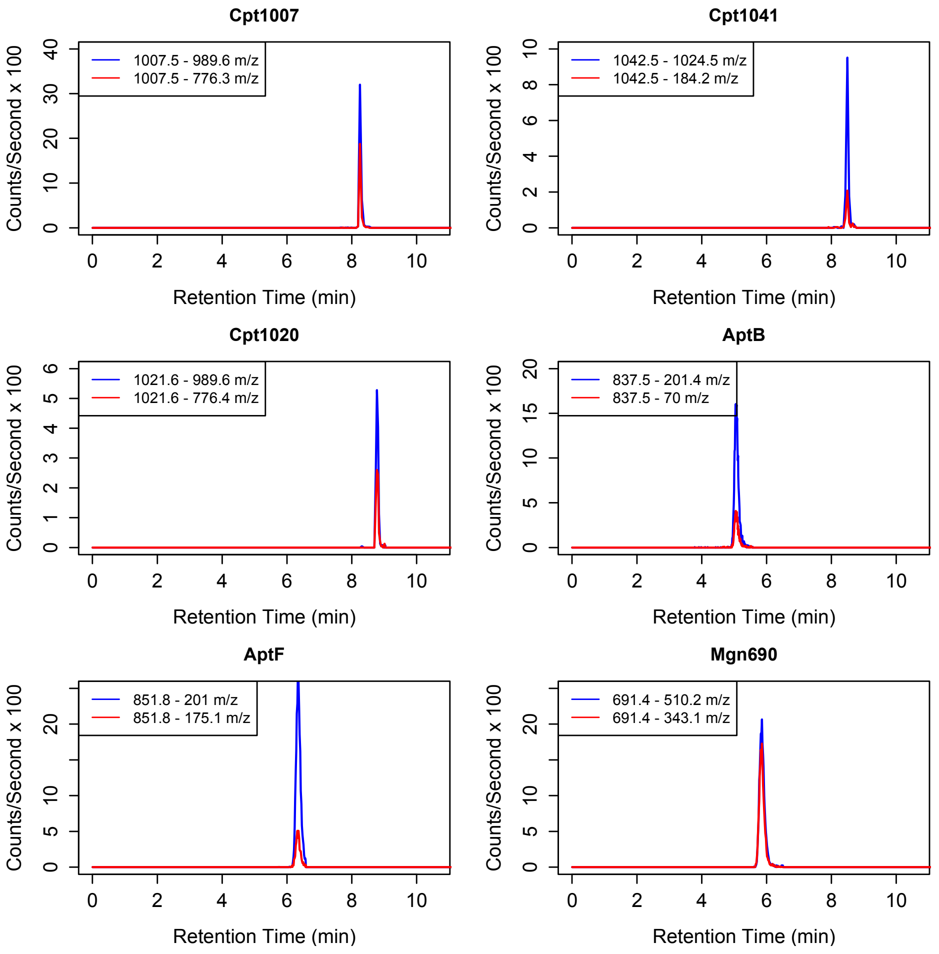

Figure S7. Chromatograms produced from a 10 µg·L^{-1} standard of cyanopeptolins (Cpt 1007/1020/1041), anabaenopeptins (AptB/F) and microginin-690 (Mgn690). Transition ions in blue were chosen for quantitation, and red transitions are confirmatory ions.

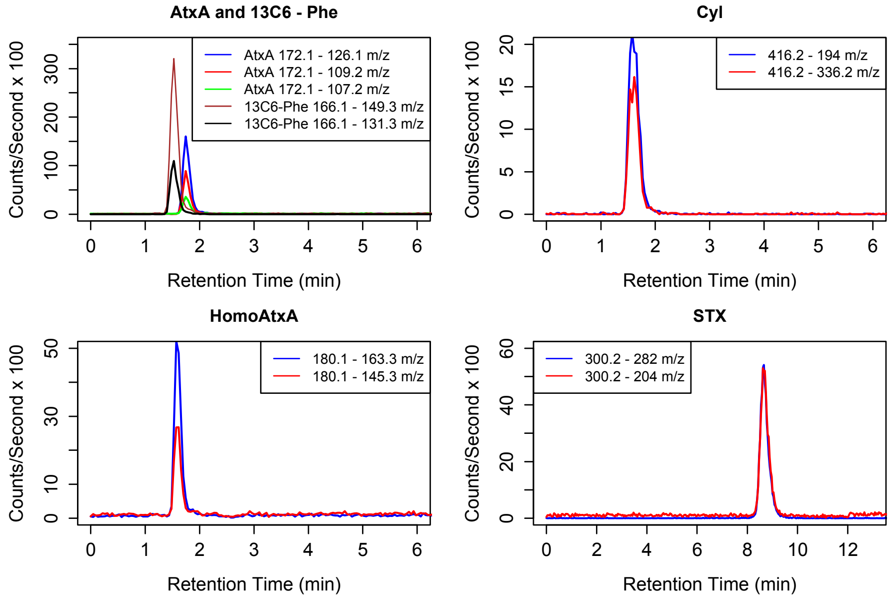

Figure S8. Chromatograms produced from a 10 µg·L^{-1} standard of anatoxin-a (AtxA), $^{13}C_6$-phenylalanine ($^{13}C_6$-Phe), cylindrospermopsin (Cyl), homoanatoxin-a (HomAtxA), and saxitoxin (STX). Transition ions in blue (or brown for $^{13}C_6$-Phe) were chosen for quantitation, and red/black transitions are confirmatory ions.
